# Development of a method for the determination of ultra-trace level mercury in adipose tissue by cold vapour atomic fluorescence spectrometry

**DOI:** 10.1155/S1463924600000146

**Published:** 2000

**Authors:** Keith E. Levine, Reshan A. Fernando, M. Lang, Amal Essader, Robert W. Handy, Bradley J. Collins

**Affiliations:** 1 Research Triangle Institute 3040 Cornwallis Road, P.O. Box 12194 Research Triangle Park> 27709-2194 NC USA; 2 National Institute of Environmental Health Sciences P.O. Box 12233 Mail Drop A2-02 Research Triangle Park 27709 USA

## Abstract

A method for the determination of total mercury in rat adipose tissue by cold vapour atomic fluorescence spectrometry (CVAFS) has been developed. Adipose samples were initially subjected to a lyophilization procedure in order to facilitate the homogenization and accurate weighing of small tissue aliquots (~50 mg). A closed vessel microwave digestion procedure using a mixture of sulphuric and nitric acids was used to liberate mercury from the adipose matrix. All mercury species were quantitatively oxidized to Hg(II) by a potassium bromate/bromide oxidation, then reduced to Hg(0) vapour by stannous chloride prior to fluorescence detection. The CVAFS exhibited a linear range of 10 pg Hg/ml to 120 pg Hg/ml. The method detection limit in solution was 2 pg Hg/ml, or 1 ng Hg/g adipose tissue, based on a nominal 50 mg sample and a final volume of 25 ml. A reference material from the National Research Council of Canada (DOLT-2, trace metals in dogfish liver) was prepared in quadruplicate in order to assess the accuracy and precision of the method. Mercury in this material was recovered at 2.22 ± 0.08 μ g/g, which is 104% of the certified level (2.14 ± 0.10 μ g/g).


Journal of Automated Methods & Management in Chemistry, Vol. 22, No. 4 (July–August 2000) pp. 103–108

### Development of a method for the determination of ultra-trace level mercury in adipose tissue by cold vapour atomic fluorescence spectrometry

**Keith E. Levine, Reshan A. Fernando, M. Lang, Amal Essader, Robert W. Handy and Bradley J. Collins[1]**

*Research Triangle Institute, 3040 Cornwallis Road, P.O. Box 12194, Research Triangle Park, NC 27709-2194, USA, [1]National Institute of Environmental Health Sciences, P.O. Box 12233, Mail Drop A2-02, Research Triangle Park, NC 27709, USA*

*A method for the determination of total mercury in rat adipose tissue by cold vapour atomic fluorescence spectrometry (CVAFS) has been developed. Adipose samples were initially subjected to a lyophilization procedure in order to facilitate the homogenization and accurate weighing of small tissue aliquots (~50 mg). A closed vessel microwave digestion procedure using a mixture of sulphuric and nitric acids was used to liberate mercury from the adipose matrix. All mercury species were quantitatively oxidized to Hg(II) by a potassium bromate/bromide oxidation, then reduced to Hg(0) vapour by stannous chloride prior to fluorescence detection. The CVAFS exhibited a linear range of 10 pg Hg/ml to 120 pg Hg/ml. The method detection limit in solution was 2 pg Hg/ml, or 1 ng Hg/g adipose tissue, based on a nominal 50 mg sample and a final volume of 25 ml. A reference material from the National Research Council of Canada (DOLT-2, trace metals in dogfish liver) was prepared in quadruplicate in order to assess the accuracy and precision of the method. Mercury in this material was recovered at 2.22± 0.08 μg/g, which is 104% of the certified level (2.14± 0.10 μg/g).*

### Introduction

The proliferation of mercury in the environment has fuelled the search for analytical methods capable of measuring trace levels of this element in a variety of sample matrices. The highly toxic nature of mercury and its tendency to accumulate in tissues has made its quantitation in biological samples of particular interest.

Numerous references detailing mercury determination in mammalian brain, liver, reproductive and kidney tissues can be found in the literature. Sample digestion schemes and modes of instrumental analysis for these methods vary greatly. Neutron activation analysis (NAA) has recently been employed to measure the level of mercury in human liver [1, 2] and brain, kidney, and lung tissues during autopsy [1]. Autometallography (AMG) was used to detect mercury in the testicular tissue of an infertile human patient [3]. The distribution of mercury in the arctic marine mammal population was studied with cold vapour atomic absorption spectrometry (CVAAS) after kidney and liver tissues were subjected to rigorous digestion procedures [4, 5]. The level of mercury in human brain and bovine liver samples was also recently measured with inductively coupled plasma mass spectrometry (ICP-MS) after the samples were subjected to microwave digestion [6].

Although an abundance of information about the determination of mercury in the above-mentioned tissues is available, relatively few methods for measuring this element in adipose tissue have been published [7]. Because adipose tissue may function as a mercury storage depot, its analysis is necessary in order to fully assess the distribution of mercury species within an organism. This is especially true for certain organic forms of mercury that tend to distribute favourably in adipose media [8].

An often overlooked mode of instrumental analysis that can be used for highly sensitive and selective mercury determinations is atomic fluorescence spectrometry (AFS). When this technique is coupled to cold vapour generation (CVAFS), the sensitivity can be greater than that observed for comparable atomic absorption techniques [9–12]. Liang and Bloom [13] successfully applied CVAFS to measure the level of mercury in biological samples in 1993.

CVAAS and CVAFS each require exhaustive sample pretreatment prior to any mercury determination in biological tissues. For both techniques, the pretreatment step must meet three prerequisites. These requirements are: (i) organic matter present in the sample must be adequately oxidized to liberate mercury from the matrix; (ii) care must be taken not to lose volatile organic mercury species; and (iii) all mercury species must be converted to Hg(II) which will eventually be reduced to Hg(0) prior to detection. A broad array of decomposition techniques has been employed to meet these objectives for biological samples. These range from room temperature alkaline procedures using tetramethylammonium hydroxide (TMAH) [14] to elevated temperature methods using combinations of mineral acids (typically $H_2SO_4$, HCl and $HNO_3$) and oxidants (typically $H_2O_2$, $KMnO_4$ and $KBrO_3$) [15–18]. Although both open and closed vessel digestion techniques have been employed to decompose biological samples at elevated temperatures, closed vessel systems are preferable as they minimize the loss of volatile organic mercury species [15]. Many of the pretreatment procedures described in the literature also utilize a sample-drying step prior to digestion. The loss of volatile (organic) mercury species that is often encountered during oven-drying procedures [15] is typically not observed when tissues are lyophilized at a lower temperature [19, 20]. As a consequence, many pretreatment methods described in the literature utilize lyophilization for sample drying [18].

In the present work, a method has been developed for the determination of trace level mercury in rat adipose tissue by cold vapour atomic fluorescence spectrometry (CVAFS). Adipose samples were lyophilized before digestion in order to facilitate the homogenization and accurate weighing of small tissue aliquots (~50 mg). Mercury was then released from the adipose matrix by a closed vessel microwave digestion in a mixture of $H_2SO_4$ and $HNO_3$. Mercury species were quantitatively oxidized to Hg(II) by a potassium bromate oxidation and reduced to Hg(0) vapour by stannous chloride prior to fluorescence detection.

### Experimental

### *Instrumentation*

Adipose tissue samples were subjected to a lyophilization procedure using a VirTis freeze dryer. This system was equipped with a 5 l condenser capacity and a Leybold Trivac® E vacuum pump. All samples were frozen at − 20 °C for ~48 h before lyophilization. Sample masses were determined using a Mettler AT 261 electronic analytical balance with a resolution of 0.1 mg over the entire 205 g weighing range, and a moving fine range (DeltaRange) with a 0.01 mg resolution.

A CEM MDS-2000 laboratory microwave system was used for sample digestion. This instrument has an operator selectable power output of 0–630 W and a direct drive alternating turntable. PrepLink version 2.0 software, installed on a Zeos 386+ notebook computer, allowed for remote operation of the MDS-2000. Adipose samples were digested in 23-ml, flat-bottomed Perfluoroalkoxy (PFA) Teflon® vials from the Savillex Corporation. These vials fit comfortably inside larger (PFA) Teflon® decomposition vessels obtained from CEM, which were employed to prevent the escape of harmful gases in the event of a vial rupture and allowed the use of the rotating turntable.

After microwave digestion, samples were quantitatively transferred into acid-cleaned volumetric flasks with deionized water. Sample preparation procedures were carried out under a high-efficiency particulate air (HEPA) filter in order to minimize potential mercury contamination. Instrumental analysis and sample aliquoting were completed under Class 10,000 conditions, while sample and standard preparation were completed in a Class 100 clean room.

Mercury data were obtained using a modular Merlin Plus atomic fluorescence spectrometer from PS Analytical. A random access autosampler (PS Analytical 20.100) was employed to deliver samples to the vapour generator (PS Analytical 10.003) where sample and reductant flows were mixed prior to a gas–liquid separator. Moisture was removed from the resulting mercury vapour with a Perma Pure MD series dryer tube and introduced into the Merlin fluorescence detector (PS Analytical 10.023). Avalon software (version 2.11), installed on a Compaq DeskPro XE 560 personal computer (Pentium, 60 MHz) was employed for both instrument control and data processing.

### *Reagents*

The acids used for sample digestion and solution preparation ($HNO_3$, HCl, $H_2SO_4$) were Fisher trace-metal grade acids and were suitable for use as purchased. The potassium bromide (EM Science, IR grade) and potassium bromate (Fisher, ACS grade) were heated in a Thermolyne Type F6000 muffle furnace at 250 °C overnight before use to drive off any mercury present. A KBr/$KBrO_3$ solution was prepared by adding separate aliquots of KBr (5.95 g) and $KBrO_3$ (1.40 g) to a 500-ml volumetric flask and diluting to volume with deionized water. Tin (II) chloride dihydrate (EM Science, analytical grade) and hydroxylamine hydrochloride (Fisher, ACS grade) were tested for their mercury background prior to use and required no additional clean-up or pretreatment. The mercury standards used for the calibration curve were prepared by dilution of a 1000 mg Hg/l (High Purity Standards) stock solution. Deionized water (18 MΩ) obtained from a Hydro Picosystem Plus deionization system was used for all solutions and labware preparations. Fisher HPLC grade acetone was used during labware cleaning. Dogfish liver (DOLT-2) from the National Research Council of Canada was used as a certified reference material for total mercury.

### *Labware preparation*

Following an adipose digestion procedure, Teflon-lined vials were soaked in phosphate-free detergent for ~4 h. After rinsing with warm tap water, any residual fat was removed with a clean room towel and acetone. Vials were then rinsed several times with tap water and were soaked overnight in a 4 N hydrochloric acid bath at room temperature. The next morning, vials were removed from the bath, rinsed several times with deionized water, and dried under HEPA filtered air. If not used immediately, the vials were sealed in plastic storage bags.

Prior to analysis, plastic autosampler cups were soaked in phosphate-free detergent, rinsed with DI water, and placed in a 50% (v/v) nitric acid bath for a minimum of 16 h. The cups were then removed from the bath, rinsed with deionized water, and dried under HEPA filtered air. Cups were sealed in plastic storage bags if not used immediately.

All other labware (Teflon® and glass) used in this investigation was soaked overnight in phosphate-free laboratory detergent. It was then rinsed copiously with tap water and leached in a 4 N hydrochloric acid bath at 70 °C for a minimum of 6 h. After removal from the acid bath, labware was rinsed with deionized water, dried under HEPA filtered air, and stored in sealed plastic bags until use.

*Table 1. Preparation of rat adipose tissue mercury matrix standards.*

| *Matrix standard* | *Number of preparations* | *Volume of 10 ng Hg/ml spiking solution (μl)* | *Spiked {Hg} in adipose tissue (pg Hg/ml)* |
|---|---|---|---|
| High concentration | 4 | 250 | 100 |
| Mid concentration-4 | 2 | 200 | 80.0 |
| Mid concentration-3 | 2 | 150 | 60.0 |
| Mid concentration-2 | 2 | 125 | 50.0 |
| Mid concentration-1 | 2 | 100 | 40.0 |
| Low concentration | 4 | 65.0 | 26.0 |
| Blank | 5 | 0.00 | 0.00 |

*Table 2. CEM MDS-2000 microwave digestion programs.*

| *Program one* | | *Program two* | |
|---|---|---|---|
| *Power (%)* | *Time (min)* | *Power (%)* | *Time (min)* |
| 10 | 1 | 20 | 1 |
| 0 | 1 | 0 | 1 |
| 10 | 1 | 20 | 1 |
| 0 | 1 | 0 | 1 |

*Sample preparation*

Rat adipose tissue from several undosed animals was pooled together in a clean container to form a control sample. The limited quantity and the fibrous and oily nature of the control tissue made mechanical homogenization difficult. As a consequence, it was impossible to obtain small (~50 mg) aliquots that were representative of the entire adipose sample. This difficulty necessitated sample pre-treatment prior to aliquoting. The control tissue was stored in a freezer at $-20\,^{\circ}C$, then lyophilized for 48 h. The dried sample allowed for easy manual homogenization with a Teflon-coated spatula. Approximately 50 mg of the homogenized rat adipose control tissue was then added to each of 21 Teflon® digestion vials. Sixteen of these aliquots were fortified with mercury as described in table 1. The remaining five unfortified aliquots served as blanks.

Adipose samples were subjected to an overnight room temperature pre-digestion step prior to microwave decomposition. A 1.5-ml volume of a 30% $H_2SO_4$/70% $HNO_3$ (v/v) acid mixture was added to each Teflon® vial. The vials were then tightly capped and placed in a Class 100 environment overnight. The following morning, samples were uncapped in order to release pressure build up. The vials were then recapped and sealed inside larger (~125 ml) Teflon® digestion vessels. These larger bombs were employed to contain harmful gases in the event of a vial rupture and also allowed the use of the rotating turntable. The samples were then subjected to a series of microwave programs presented in table 2 (program one was run four times and program two was run twice).

Upon completion of the microwave procedure, sample vials were removed from the larger Teflon® vessels and allowed to cool to room temperature in a Class 100 environment. These vials were vented and 1.5 ml each of HCl and a $KBr/KBrO_3$ solution was then added to each sample. The vials were sealed and allowed to oxidize overnight at room temperature. The following morning, a 150-μl aliquot of a 12% (w/v) $NH_2OH$ HCl solution was added to each Teflon® digestion vial to remove any excess bromine from the oxidized samples. Each adipose sample was then quantitatively transferred to a clean 25 ml volumetric flask and diluted to volume with deionized water.

*CVAFS analysis*

Samples were analysed by CVAFS immediately upon completion of the bromate–bromide oxidation procedure. The instrument was calibrated with mercury standards ranging in concentration from 10 pg/ml to 120 pg/ml. Calibration standards were prepared on the day of analysis in a solvent that approximated the composition of the samples: 6% (v/v) $H_2SO_4/HNO_3$, 6% (v/v) HCl, 6% (v/v) $KBr/KBrO_3$ and 0.6% (v/v) $NH_2OH$ HCl. The CVAFS instrumental parameters are presented in table 3.

Several quality control (QC) measures were taken during the analysis of samples. A check blank and a mid-level calibration standard were run before and after each group of 10 samples analysed. The per cent error of the QC check standard was required to be within 10% of its nominal concentration for the analysis of bracketed samples to be considered valid. In addition, several method blanks and certified reference materials were analysed in order to assess potential Hg contamination and method accuracy.

### Results and discussion

Data for the CVAFS determination of mercury in rat adipose matrix standards are presented in table 4. The instrument was calibrated with mercury standards prior to sample analysis. A least squares linear regression equation was calculated from these standards as $y = 1.393(x) - 0.4306$, where '$y$' is the intensity of the fluorescence response (peak height) and '$x$' is the mercury concentration expressed in pg Hg/ml. When the responses obtained from the calibration standards were compared against those obtained from the matrix standards prepared at identical concentrations, a suppression of the mercury signal was observed. Mercury spikes in the rat adipose matrix were recovered from 70.3% to 73.7%

*Table 3. CVAFS instrumental parameters.*

| | |
|---|---|
| Detector: | Merlin (Hg-specific) fluorescence |
| Excitation/emission wavelength: | 254 nm |
| Detector fine gain: | 10 |
| Detector coarse gain: | 10 |
| Zero: | Auto |
| Curve fit: | Least squares linear fit |
| Measured by: | Peak height |
| Delay time: | 5 s |
| Rise time: | 25 s |
| Analysis time: | 30 s |
| Memory time: | 60 s |
| Gas–liquid separator: | Mercury type |
| Reductant: | $SnCl_2$ |
| Blank: | ($H_2SO_4/HNO_3$, HCl, KBr/$KBrO_3$, $NH_2OH$ HCl) |
| Reductant flow rate: | 2–5 ml/min |
| Blank flow rate: | 7–10 ml/min |
| Sample flow rate: | 7–10 ml/min |
| Carrier gas (argon): | 0.4 l/min |
| Sheath gas (argon): | 0.3 l/min |
| Dryer gas (argon): | 3 l/min |

*Table 4. Mercury recoveries in rat adipose tissue matrix.*

| Nominal {Hg} (pg/ml) | Number of preps. | Avg. response of matrix stds. (peak height) | Response precision (% RSD) | Cal. curve response (peak height) | Recovery (%)[b] |
|---|---|---|---|---|---|
| 0.00 | 5 | 9.600 | 10 | 0.350 | NA[a] |
| 26.0 | 4 | 35.10 | 2.3 | 35.79 | 72.0 |
| 40.0 | 2 | 48.15 | 7.2 | 53.65 | 72.3 |
| 50.0 | 2 | 58.00 | 0.24 | 69.22 | 70.3 |
| 60.0 | 2 | 66.89 | 2.6 | 81.90 | 70.3 |
| 80.0 | 2 | 88.04 | 3.1 | 110.9 | 71.0 |
| 100 | 4 | 110.7 | 0.49 | 137.6 | 73.7 |

[a] NA, not applicable.

[b] Calculated as blank corrected (matrix response/calibration curve response) 0 × 100%.

for digest concentrations ranging from 26.0 pg Hg/ml to 100 pg Hg/ml.

The narrow recovery range of the adipose matrix curve suggests that a matrix correction factor could accurately be applied to a regression equation calculated from calibration standards. To illustrate this point, the mercury response (peak height) in the rat adipose matrix was plotted against the concentration (pg Hg/ml) of mercury added to each matrix standard replicate (figure 1). The least squares linear regression equation calculated from these data was $y = 1.021(x) + 7.744$, where '$y$' and '$x$' are as described above. When the slope of this adipose matrix curve is divided by the slope of the solvent curve, a factor of 0.7330 results. The linearity of the adipose matrix curve ($r = 0.9983$) allows for this correction factor to be applied to the calibration curve, eliminating the need for time-consuming standard additions procedures.

The per cent relative standard deviation (%RSD) for replicate preparations of spiked mercury in the adipose matrix ranges from 0.24 to 7.20% (table 4). This level of precision indicates adequate homogenization of the control adipose matrix. After lyophilization, it was easier to manually homogenize the control sample and accurately remove small aliquots.

A suitable certified reference material (CRM) was available, so a check for method accuracy was performed. A sample of DOLT-2 (trace metals in dogfish liver tissue) was analysed in quadruplicate 50-mg aliquots. The mean

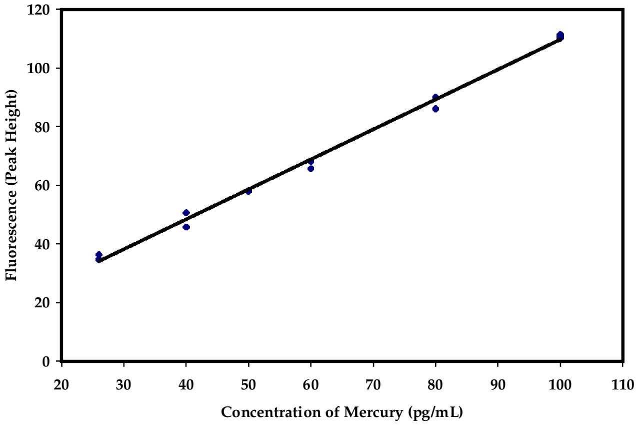

*Figure 1. Mercury response curve in the rat adipose matrix.*

*Table 5. Biological sample analysis of adipose tissue obtained from dosed and control animals.*

| *Hg vapour dosing level (mg/m$^3$)* | *Time point* | *Measured {Hg} in control animals (ng/g)* | *Measured {Hg} in dosed animals (ng/g)* |
|---|---|---|---|
| 1 | GD6 | < 1–1.905 | 9.802–34.38 |
| | GD10 | < 1–1.905 | 18.80–36.99 |
| | GD15 | < 1–1.905 | 12.11–31.92 |
| 2 | GD6 | < 1–5.581 | 47.23–86.15 |
| | GD10 | < 1–5.581 | 54.43–131.3 |
| | GD15 | < 1–5.581 | 51.74–83.67 |
| 4 | GD6 | all samples < 1 | 108.7–220.3 |
| | GD10 | all samples < 1 | 134.5–302.0 |
| | GD15 | all samples < 1 | 232.1–526.1 |
| 8 | GD6 | < 1–1.566 | 115.0–270.1 |
| | GD10 | < 1–1.566 | 498.1–1094 |
| | GD15 | < 1–1.566 | 1504–1851 |

concentration of total mercury in these samples was 2.22 ± 0.08 g/g, which was 104% of the certified level (2.14 ± 0.10 g/g). These results indicate good accuracy, precision and freedom from interferences from other metals present in the CRM.

### *Detection limits*

The method detection limit (MDL) was calculated as three times the standard deviation of the calculated values for the lowest concentration matrix standards (26.0 pg/ml). The resulting mercury MDL for the analytical method was 2 pg Hg/ml. This corresponds to 1 ng Hg/g of adipose tissue, based on an initial sample mass of 50 mg and a volume of 25 ml. The method quantitation limit (MQL) was calculated as 10 times the standard deviation of the calculated values for the lowest concentration matrix standard. The resulting MQL for the adipose matrix was 8 pg Hg/ml or 4 ng Hg/g adipose tissue, based on a sample mass of 50 mg and a volume of 25 ml.

### *Application of method*

The utility of the developed method was tested through the analysis of samples obtained from rats in a mercury vapour dose–response study. A summary of data collected from control ($n = 20$) and dosed animals ($n = 60$) is presented in table 5. Animals were sacrificed at several time points for each dosing level. The level of mercury found in the adipose tissue of control animals was generally less than the MDL for all time points. The range of mercury measured in the adipose tissue of dosed animals was proportional to both the vapour dosing level and the death time point. This trend is illustrated as a histogram in figure 2.

Mercury concentration data presented in table 5 were calculated using the mass of adipose tissue before lyophilization. In order to assess the amount of water removed from adipose tissue during freeze-drying, several samples ($n = 26$) were weighed both before and after the lyophilization procedure. The per cent weight loss on drying (%LOD) was calculated as (g total wet mass − g total dry mass) ÷ g total wet mass. The average %LOD for the samples was 13.3 ± 3.25%, with values ranging from 7.72 to 18.9%.

### Conclusion

The analysis of adipose tissue is necessary in order to fully assess the distribution of mercury within a biological organism. In the present work, a method for the determination of ultra-trace level mercury in adipose tissue by CVAFS was developed. The sensitivity and selectivity of CVAFS allows for the determination of ultra-trace level mercury in small sample masses (~50 mg) which is advantageous in instances where a high level of sensitivity is required and sample size is limited. The small aliquot size also simplifies the closed vessel microwave digestion procedure by reducing the build up of decomposition gases. Sample homogenization was necessary in order to ensure that the small adipose aliquots were representative of the entire sample. A lyophilization procedure was employed to help homogenize adipose samples prior to analytical aliquot removal.

The developed method was put to practical use through the analysis of samples obtained from rats in a mercury vapour dose–response study. The level of mercury determined for undosed animals was generally less than the MDL of 1 ng Hg/g adipose tissue. The concentration of mercury found in the adipose tissue of dosed animals was proportional to both the mercury dose level and the length of time between exposure and death.

A potential drawback of the described method is the time required to complete an analysis procedure. A batch of ~50 adipose samples and controls can be prepared and analysed over a 5-day work week. This disadvantage is offset somewhat by the low labour investment required for many of the sample preparation procedures. For example, the lyophilization, many of the digestion steps, and the FI-CVAFS analysis are automated and do not require constant monitoring by laboratory personnel.

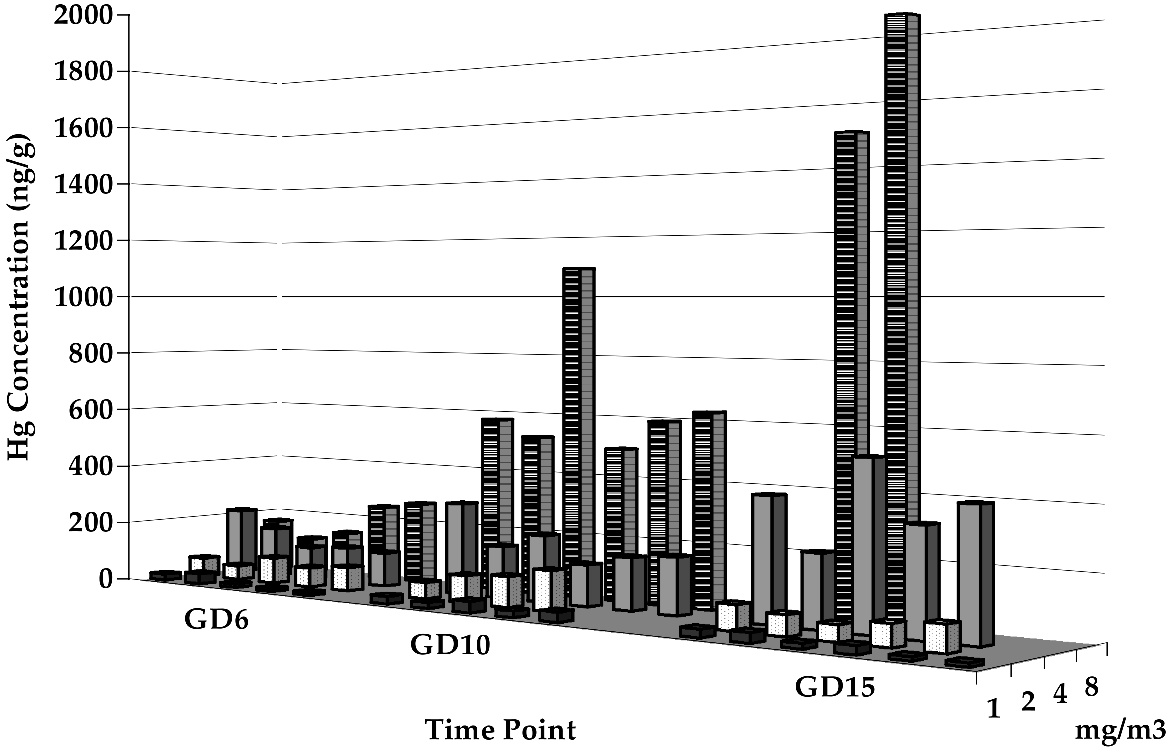

*Figure 2. Mercury concentration found in the adipose tissue of dosed animals.*

### Acknowledgement

This project has been funded in whole from the National Institute of Environmental Health Sciences, National Institutes of Health, Contract Number: N01-ES-55386.

### References

1. Barregård, L., Sällsten, G. and Conradi, N., *Int. Arch. Occup. Environ. Health*, **72** (1999), 169.
2. Goeij, J. J. M., Volkers, K. J. and Tjioe, P. S., *Analytica Chimica Acta*, **109** (1979), 139.
3. Keck, C., Bergmann, M., Ernst, E., Müller, C., Kliesch, S. and Nieshlag, E., *Reproductive Toxicology*, **7** (1993), 469.
4. Wagemann, R., Innes, S. and Richard, P. R., *The Science of the Total Environment*, **186** (1996), 41.
5. Zeisler, R., Demiralp, R., Koster, B. J., Becker, P. R., Burow, M., Ostapczuk, P. and Wise, S. A., *The Science of the Total Environment*, **139/140** (1993), 365.
6. Krachler, M., Radner, H. and Irgolic, K. J., *Fresenius Journal of Analytical Chemistry*, **355** (1996), 120.
7. Wagemann, R., Trebacz, E., Boila, G. and Lockhart, W. L., *The Science of the Total Environment*, **218** (1998), 19.
8. Ferens, M. C., *A Review of the Physiological Impact of Mercurials*, Project Officer: Holm, H. W. (U.S. Environmental Protection Agency) EPA-660/3-73-022 (1974).
9. West, C. D., *Analytical Chemistry*, **46** (1974), 797.
10. Thompson, K. C. and Godden, R. G., *Analyst*, **100** (1975), 544.
11. Stockwell, P. B. and Corns, W. T., *Journal of Automatic Chemistry*, **15** (1993), 79.
12. Stockwell, P. B., *Laboratory Practice*, **39** (1990), 29.
13. Liang, L. and Bloom, N. S., *Journal of Atomic Absorption Spectrometry*, **8** (1993), 591.
14. Tao, Y., Willie, S. N. and Sturgeon, R. E., *Analyst*, **123** (1998), 1215.
15. Van Delft, W. and Vos, G., *Analytica Chimica Acta*, **209** (1988), 147.
16. Iskandar, I. K., Syers, J. K., Jacobs, L. W., Keeney, D. R. and Gilmour, J. T., *Analyst*, **97** (1972), 388.
17. Murphy, J., Jones, P. and Hill, S. J., *Spectrochimica Acta B*, **51** (1996), 1867.
18. De Vargas, M. C. and Romero, R. A., *Atomic Spectroscopy*, **10** (1989), 160.
19. Ramelow, G. and Hornung, H., *Atomic Absorption Newsletter*, **17** (1978), 59.
20. Lafleur, P. D., *Analytical Chemistry*, **45** (1973), 1534.

